# Trends in toxicological findings and drug seizures of MDMA in New Zealand from 2010 to 2022

**DOI:** 10.1111/1556-4029.70284

**Published:** 2026-02-12

**Authors:** Thomas J. Sheehan, Hilary J. Hamnett, Sarah G. G. Russell, Diana Kappatos, Helen Poulsen

**Affiliations:** ^1^ PHF Science Porirua New Zealand; ^2^ Department of Chemistry University of Lincoln Lincoln UK

**Keywords:** 3,4‐methylenedioxymethamphetamine, driving under the influence, drug seizure, ecstasy, MDA, MDMA, postmortem, trends

## Abstract

3,4‐Methylenedioxymethamphetamine (MDMA) or “ecstasy,” is a commonly used drug worldwide, including in New Zealand, where 4.8% of the population aged 15 years or over reported using it in a 2023/2024 survey. This study provides additional insights on MDMA prevalence data in New Zealand by reporting toxicology data from Coronial and driving under the influence of drugs (DUID) cases positive for MDMA between 2010 and 2022. Presented alongside are data from MDMA drug seizures (tablets, powders/crystals, and capsules) submitted by NZ Police or Customs during the same period. Of the 131 MDMA‐positive Coronial cases, 73 were quantified in peripheral postmortem blood (mean: 0.88 mg/L, range: 0.01–9.30 mg/L). Of the 193 DUID cases positive for MDMA, 186 were quantified in blood (mean: 0.23 mg/L, range: 0.01–7.30 mg/L). MDA was also quantified in peripheral blood in 33 Coronial cases (mean: 0.06 mg/L, range: 0.01–0.20 mg/L) and 79 DUID cases (mean: 0.03 mg/L, range: 0.01–0.21 mg/L). In both types of MDMA‐positive cases, 85% or more were positive for other drugs and/or alcohol, with the most commonly co‐used substances being cannabis and alcohol. The demographics of MDMA users were comparable to those reported in previous studies. The prevalence of MDMA in drivers (1.8%) is similar to that reported in previous studies. There were 138 drug seizures with an average purity of 71% (range: 17–101%), with capsules showing the highest overall purity. We envisage the data in this paper being used by forensic toxicologists, law enforcement and drug policy makers.

**Highlights**

- MDMA prevalence in drivers in New Zealand is similar to previous studies.
- Co‐use of other drugs and/or alcohol is common in MDMA cases (found in ≥85%).
- The most commonly co‐used substances were cannabis and alcohol.
- Peripheral blood MDMA and MDA concentrations were higher in Coronial compared to DUID cases.
- MDMA capsules in New Zealand had a higher purity than pills or powders/crystals.

## 1 INTRODUCTION

3,4‐Methylenedioxymethamphetamine (MDMA), commonly known as “ecstasy,” is a drug that has been commonly used in New Zealand (NZ) since the late 1980s [1], with the first National Drug Survey in 1998 finding that 1.5% of the population had used ecstasy in the past year [2]. This phenethylamine stimulant is often assumed as a “party drug” and has been found in recent NZ studies to be in high use at summer music festivals [3]. MDMA is also well known in the clubbing scene internationally [4] for its central nervous system stimulation, euphoric, and empathetic effects [5]. Recent survey data from NZ indicates that 3.6% of the population aged 15 and over reported using MDMA in 2022/2023 [6] and 4.8% in 2023/2024 [7]. This is high relative to a global estimate of 0.4% in the 2023 World Drug Report [8] and Australia and NZ are the highest per capita users of MDMA in the world [9]. MDMA use appears to be concentrated among younger people (15–24‐year olds) [7] and wastewater testing suggests it is more prevalent in both the South Island of NZ and in major urban centers [10]. There is also some evidence that MDMA's availability in NZ is decreasing [7] potentially due to import barriers; MDMA is not made within NZ but imported from overseas [11]. MDMA is a Class B controlled drug under the Misuse of Drugs Act 1975 legislation in NZ.

MDMA use can lead to hospitalization [12] and even fatalities [13] typically due to complications arising from hyperthermia. There are also other health risks associated with the use of MDMA such as the adulterants added to MDMA pills [14], the use of “ecstasy” pills that actually contain other drugs such as novel psychoactive substances that may be more toxic, and the trend toward high‐dose pills (>100 mg MDMA per pill) [15]. MDMA can also cause driving impairment [16] by affecting information processing, coordination, and concentration [17], and is often detected in drivers who have been involved in motor vehicle crashes (MVCs) [18, 19]. In a study of drivers who had been hospitalized following a crash in NZ between October 1, 2019 and January 31, 2020, 4% tested positive for MDMA [20].

While drug use surveys provide valuable insights into the prevalence of MDMA, they rely on self‐reported data and can be subject to sampling bias [21]. Similarly, wastewater testing can examine MDMA use at the population level, but cannot provide information on prevalence or purity. This study aims to examine MDMA trends in NZ using multiple data sources including deaths, driving under the influence of drugs (DUID) cases, and drug seizures submitted by NZ Police or Customs. Detailed toxicological data are essential for forensic toxicologists who are interpreting MDMA concentrations in casework. As part of this study, we also aimed to provide recent MDMA and 3,4‐Methylenedioxyamphetamine (MDA) concentrations in Coronial and DUID cases. MDA is the main active metabolite of MDMA, as well as a drug in its own right. Although MDA can be routinely analyzed in forensic toxicology, there is limited published data on its concentrations in postmortem and DUID cases where MDMA is also detected. As an additional dataset, MDMA‐containing drug samples submitted by NZ Police or Customs have also been analyzed.

## 2 METHODS

This is a retrospective study of MDMA‐positive toxicology cases and drug samples analyzed by PHF Science in NZ over the period 2010 to 2022. Data for this study were obtained from three sources: DUID cases, deaths referred to the Coroner, and MDMA‐containing drug samples from NZ Police or Customs seizures. All DUID and Coronial cases requiring toxicology testing in NZ are sent to the PHF Science Toxicology laboratory in Wellington. Drugs cases are analyzed by the PHF Science Drugs laboratory in Auckland. The population covered by these forensic laboratories is c. 5 million people, according to the 2023 NZ Census [22].

### 2.1 DUID case selection

Driving under the influence cases are prosecuted under the Land Transport Act 1998 (LTA) legislation in NZ. Samples received under the LTA from January 2010 to December 2022, which were analyzed for evidence of drug use and were positive for MDMA, were included in this study. These blood samples were from two types of drivers:

1. Those who were stopped due to poor driving passed a roadside breathalyzer test for alcohol but failed a compulsory impairment test and were subsequently charged with “Driving under the influence of drugs and incapable of proper control” under section 58(1) of the LTA (for first or second offenses) or “Driving while impaired by drugs” under section 57D (third offenses and beyond).
2. Those who were involved in a motor vehicle crash (MVC) and hospitalized as a result. Drivers who are involved in a MVC, but are not themselves injured, are breathalyzed but not routinely tested for drugs unless they pass the breathalyzer test but still appear impaired.

Drivers cannot be stopped randomly for potential drug‐driving offenses under the current NZ legal framework (NZ Police require “just cause” to stop a driver), although as of December 2025 this will change.

The DUID cases selected for this study are those where drugs analysis had been requested. This does not encompass all DUID cases received by our laboratory. There are instances where only alcohol testing is requested, for example, in a hospitalized driver who was unable to be breathalyzed at the roadside. Requests for analysis of blood samples from hospitalized drivers are often limited to alcohol only, but on request from NZ Police, analyses can be extended to incorporate drugs, including MDMA. If the blood from a hospitalized driver has alcohol above the legal limit, further analysis for other drugs is only requested in a few cases. Another instance is where a driver who has been breathalyzed is over the alcohol limit, and requests a blood sample be taken for laboratory analysis. These blood samples are not analyzed for other drug use. It should be noted that the roadside breathalyzer test is largely accepted in NZ, and relatively few drivers with alcohol above the legal limit on a breathalyzer test will ask for their blood to be analyzed.

Over the period covered by this study, there was no per se limit for MDMA in NZ for drivers and no use of roadside preliminary tests, for example, oral fluid testing for MDMA, although these will be introduced in December 2025.

### 2.2 Coronial case selection

All cases received as part of Coronial Investigations from January 2010 to December 2022 where the blood was positive for MDMA were included in this study. This includes cases where the cause of death was deemed initially to be MDMA toxicity, but also other causes of death. Not all cases referred to the Coroner will be sent for toxicological screening, with the decision to request toxicology normally lying with the pathologist carrying out the autopsy. Manner of death was not reported for these cases to the laboratory.

### 2.3 Toxicological analysis

Blood samples were submitted in vacuum‐sealed gray‐top vacutainer tubes (2% sodium fluoride and 0.5% potassium oxalate) for DUID cases. For postmortem cases, blood samples were submitted in silanized glass McCartney bottles containing 2% sodium fluoride and 0.5% potassium oxalate.

#### 2.3.1 Screening

Prior to 2013, screening of blood samples for Coronial and DUID cases was carried out using an ELISA method for MDMA/methamphetamine.

After 2013, screening and confirmation was by assisted liquid–liquid extraction (LLE) using 1:1:1 dichloromethane: dichloroethane: heptane with sodium chloride and sodium carbonate with reconstitution in mobile phase (980 mL DI water with 20 mL acetonitrile and 1 mL conc. formic acid). This was followed by a validated liquid chromatography quadrupole time‐of‐flight mass spectrometry (LC‐QTOF/MS/MS) method for c. 200 compounds targeting a wide range of medicinal and controlled drugs [23] using a Bruker Maxis QTOF/MS/MS coupled to a Dionex Ultimate 3000 ultra‐high performance liquid chromatograph (UHPLC).

#### 2.3.2 Quantification

Sample preparation for confirmation (where ELISA was the screening method) and quantification of MDMA and MDA in Coronial blood samples until 2017 was by basic LLE with sodium hydroxide and butyl chloride, reconstituted in 75:25 acetonitrile: 100 mM ammonium formate +0.5% formic acid.

In 2017, a modified QuEChERS (Quick, Easy, Cheap, Effective, Rugged, and Safe) extraction was introduced involving cold acetonitrile and QuEChERS salt, with reconstitution in 0.1% formic acid.

Across the time period of the study (2010–2022), all quantifications for both case types followed a validated liquid chromatography triple quadrupole mass spectrometry (LC–MS/MS) method on a Sciex triple quadrupole 5500 coupled to a Sciex Exion with a 7‐point standard calibration curve (range: 0.01–1.00 mg/L for both MDA and MDMA) using MDMA‐d5 as the internal standard. The method was validated according to the EU Commission concerning the performance of analytical methods and interpretation of results (2002) [24]. This method also quantifies MDA. The limit of detection (LOD) for both MDMA and MDA was 0.005 mg/L, determined experimentally during method validation.

### 2.4 Drug seizure analysis

A total of 191 tablets, capsules, or powder/crystal samples, seized either crossing the NZ border or by NZ Police during 2018/2019, and submitted by NZ Police or Customs in 2019 to the PHF Science Drugs team, were analyzed. This was a small subsample of all suspected MDMA samples seized by NZ Police or Customs during that period. Samples underwent initial analysis using Fourier transform infrared (FTIR) spectroscopy and gas chromatography mass spectrometry (GC–MS) to identify the presence of MDMA. The breakdown of sample types was *n* = 36 tablets, *n* = 105 powders/crystals, and *n* = 50 capsules.

Tablet and capsule samples were prepared by grinding in a mortar and pestle. For each sample, a c. 10 mg subsample was taken and extracted with 1.5 mL methanol and sonicated for 10 min. Where the sample size allowed, up to three subsamples were taken. Samples were further diluted with methanol to a concentration of c. 1 mg/L MDMA, assuming 100% purity. A 1 mL volume of sample was transferred to an HPLC vial and 50 μL of MDMA‐d5 internal standard was added at a concentration of 12 mg/L to give a final concentration of 0.6 mg/L. The samples were then analyzed quantitatively to determine the dose range and percentage purity of the various types of products.

Quantification of MDMA in drug seizures submitted by NZ Police or Customs was by the LC–MS/MS method described in Section 2.3.2 (but with an experimentally determined calibration range of 0.12–1.20 mg/L to include a range of 12% to 120% purity). The method was not revalidated for seized drug samples, but the calibration curve underwent verification using separate MDMA check standards. MDA was included in the scope of the method and an MDA check standard was also used. The percentage purity of the seized drug samples was determined by calculating the mass (mg) of MDMA from the measured concentration (mg/L) for each subsample, taking into account the dilution factor, then dividing by the mass of the original sample and multiplying by 100.

### 2.5 Statistical analysis

All data were transferred to MS Excel and descriptive statistics were calculated according to Desharnais [25]. Mean values for Coronial versus DUID cases were compared by a two‐sample one‐tailed unequal variance Student's independent *t*‐test. ANOVA analyses were performed comparing MDMA concentration and year, and MDMA purity and drug sample type in BioRender. Results were considered statistically significant if *p* < 0.05. Graphs were drawn using R version 4.5.0 (2025‐04‐11) (ggplot2 version 3.5.2) or BioRender.

## 3 RESULTS

### 3.1 Demographics of toxicology cases

Of the 131 Coronial cases, the ethnicity of the deceased was recorded in 107. The majority (*n* = 71, 66%) of deceased were NZ European (White) followed by Māori (*n* = 24, 22%), Pacific Peoples (*n* = 8, 7%), and Asian (*n* = 4, 4%). The first three categories were broadly in line with the 2023 NZ Census data [22]; however, Asian ethnicity was underrepresented in the Coronial data (4% compared to 17% in the Census). Ethnicity was not recorded for the DUID cases.

Gender was recorded for all 131 Coronial cases, with males being overrepresented at 74% (*n* = 97) compared to 49.3% in the Census, and females being underrepresented at 26% (*n* = 34) compared to 50.3% in the Census [22]. Gender was not recorded for the DUID cases.

The age distributions of the Coronial cases and DUID cases are shown in Figure S1, and compared to the Census. As might be expected, DUID cases are clustered around the younger age groups, with the age ranges 15–19, 20–24, 25–29, and 30–34 years overrepresented compared to the Census data. For the Coronial cases, a similar pattern is seen, but the overrepresentation extends into the 35–39 and 40–44 age ranges. The age ranges observed in this study were 16–70 years (mean: 31 ± 12.9, median: 28) for Coronial cases, and 15–66 years (mean: 28 ± 9.9, median: 25) for DUID cases. Note that age data was not available in two Coronial cases and four DUID cases.

### 3.2 Coronial cases

#### 3.2.1 Numbers of MDMA‐positive Coronial cases

Between 2010 and 2022, 23,261 Coronial cases were referred for toxicology testing to our laboratory. Of these, 131 tested positive for MDMA (0.5%). Peripheral blood was obtained at the time of autopsy in 111 cases, antemortem blood in 11 cases and postmortem blood from other sites in nine cases.

#### 3.2.2 Trends in MDMA‐positive Coronial case numbers

The overall trend in prevalence of Coronial cases over the time period is shown in Figure S2. From 2010 to 2022, the number of Coronial cases referred to the laboratory steadily increased from 1511 in 2010 to 2539 in 2022. Figure S2 shows the two spikes in MDMA‐positive cases, one in 2018 (*n* = 26) and one in 2020 (*n* = 30) followed by a sharp decline in 2021.

#### 3.2.3 Causes of death

The distribution of causes of death for the 131 MDMA‐positive Coronial cases is given in Figure S3, although for *n* = 18 cases (13.7%) the cause was “Unascertained.” “MDMA toxicity” was determined to be the cause of death in five cases (3.8%).

#### 3.2.4 Concentrations of MDMA in Coronial cases

Of the 131 Coronial cases that were positive for MDMA during the study period, the concentration of MDMA was quantified in 73. The reasons for MDMA not being quantified in every case included poor sample condition (e.g., putrefied, oily, clotted, containing solid tissue, etc.), limited sample volume, or a known cause of death, for example, drowning, MVC, etc. where MDMA quantification was not requested by the pathologist. In three cases, MDMA was present but at a concentration < LLOQ (0.01 mg/L). Results are shown in Table 2. The mean concentration of MDMA in the Coronial cases was 0.88 mg/L (range: 0.01–9.30, median: 0.20 mg/L). For the five cases determined to be due to MDMA toxicity, the MDMA concentrations were 0.02, 0.7, 2.4, 3.9, and 6.1 mg/L.

The concentrations of MDMA in Coronial cases by year are shown in Figure S4 and the average value for each year is shown in Table S1. There is an overall increase in average MDMA concentration in the Coronial cases between 2017 and 2022. The two‐way ANOVA analysis showed a significant difference in MDMA concentrations between 2017 and 2022 (*p* = 0.0272) and between 2018 and 2022 (*p* = 0.0082) for the Coronial cases.

Of the 73 Coronial cases where MDMA was quantified, MDA was also quantified in 33. Due to the small number of quantifications in each cause of death category, the data has been grouped as “Coronial” cases, rather than breaking them down. The concentrations are shown in Table 3 along with the mean ratios of MDMA‐to‐MDA. The mean concentration of MDA in the Coronial cases was 0.06 mg/L (range: 0.01–0.20, median: 0.04 mg/L). For the five MDMA toxicity cases, the MDA concentration was measured in four (0.03, 0.06, 0.2, and 0.2 mg/L), giving MDMA‐to‐MDA ratios of 40, 31, 20, and 23.

#### 3.2.5 Co‐use of other drugs in Coronial cases

In the MDMA‐positive Coronial cases, other drugs and/or alcohol were detected in 86% (*n* = 113). The mean number of co‐used drugs in Coronial cases was 2.1 (range: 1–6, median: 2.0). The most commonly co‐used substance was alcohol (*n* = 63) followed by cannabinoids (*n* = 46) and methamphetamine (*n* = 29), as reported in Figure S5 (bottom). Of the 63 Coronial cases where alcohol and MDMA were detected, only 16 had used no other drugs. The mean alcohol concentration was 125 ± 79 mg/100 mL (range: 12–416, median: 106 mg/100 mL).

### 3.3 DUID cases

#### 3.3.1 Number of MDMA‐positive DUID cases

The prevalence of MDMA in DUID cases was 1.8% (*n* = 193 out of 10,840 DUID cases). Table 1 shows a breakdown of the data for each year.

**TABLE 1:** Total number of MDMA‐positive cases between 2010 and 2022.

Year	Total Coronial cases	MDMA‐positive Coronial cases	DUID cases	MDMA‐positive DUID cases
2010	1511	1	362	0
2011	1500	0	214	0
2012	1500	1	272	0
2013	1474	3	300	0
2014	1553	2	284	3
2015	1517	1	508	1
2016	1651	6	776	0
2017	1881	10	946	12
2018	1890	26	1169	27
2019	2074	18	1523	45
2020	2012	30	1538	58
2021	2159	14	1492	29
2022	2539	19	1456	18
Total	23,261	131	10,840	193

#### 3.3.2 Trends in MDMA‐positive DUID case numbers

The overall trend in prevalence of DUID cases over the time period is shown in Figure S6. The case numbers shown represent absolute numbers of cases analyzed, not a rate based on population. From 2017 to 2022, the number of DUID cases referred to the laboratory increased from 946 to around 1500, where it has stayed consistently. Figure S6 shows a peak of 58 cases positive for MDMA in 2020, followed by a sharp decline in 2021 and 2022. This was the period affected by COVID‐19 (see Figure S6), and although the number of DUID cases remained consistent, the number of MDMA‐positive cases declined steeply.

#### 3.3.3 Concentrations of MDMA in DUID cases

Of the 193 DUID cases that were positive for MDMA during the study period, the concentration of MDMA was quantified in 186. Results are shown in Table 2. For the DUID cases, the mean MDMA concentration was 0.23 mg/L (range: 0.01–7.30, median: 0.08 mg/L). The difference between the mean concentration for Coronial and DUID cases was statistically significant (see Table 2).

**TABLE 2:** Concentrations of MDMA present in blood by case type (mg/L).

Case type	No. of cases	Min.	Max.	Mean^b^	SD	%RSD	Median
Unascertained	16	0.03	9.30	1.74	2.58	148.3	0.35
Drowning	6	0.02	1.50	0.61	0.70	115.2	0.30
GSW	2	0.01	0.02	0.02	0.01	47.1	0.02
Hanging	14	0.01	1.00	0.20	0.29	141.9	0.09
MDMA toxicity	5	0.02	6.10	2.62	2.46	93.8	2.40
MVC	12	0.01	1.60	0.57	0.58	100.9	0.40
Trauma	2	0.06	0.30	0.18	0.17	94.3	0.18
Mixed drug toxicity	10	0.01	3.10	0.66	1.12	169.6	0.06
Other^a^	6	0.05	1.00	0.46	0.43	94.2	0.30
Total Coronial	73	0.01	9.30	0.88	1.59	181.3	0.20
Hospitalized drivers	116	0.01	7.30	0.22	0.69	316.7	0.08
Impaired	70	0.01	1.70	0.24	0.32	131.7	0.09
Total DUID	186	0.01	7.30	0.23	0.57	254.7	0.08

*Note*: Blood sampling sites were peripheral *n* = 111 cases, antemortem blood *n* = 11 cases and postmortem blood from other sites *n* = 9.

Abbreviations: GSW, gunshot wound; MVC, motor vehicle crash.

^a^ Fall, fire, heart, medical cause, suspicious death, and tramadol toxicity.

^b^ A significantly higher mean concentration of MDMA was observed in Coronial cases compared to DUID cases (*p* = 0.0005).

The concentrations of MDMA in DUID cases by year are shown in Figure S4, and the average value for each year is shown in Table S1. There is no obvious trend in MDMA concentration in the DUID cases over time, and the two‐way ANOVA showed no significant differences in concentrations between years.

Of the 186 DUID cases where MDMA was quantified, MDA was also quantified in 79. In all but one, MDA was present as a metabolite of MDMA. MDA itself had apparently been used by one driver, shown by an MDMA‐to‐MDA ratio of 0.07 and an MDA concentration of 0.41 mg/L (compared to an MDMA concentration of 0.03 mg/L). This case has been excluded from Tables 3, 5, 6 and 7. The mean MDA concentration in the DUID cases was 0.03 mg/L (range: 0.01–0.21, median: 0.02 mg/L excluding the outlier).

The difference between the mean MDA concentrations for Coronial and DUID cases was statistically significant (see Table 3). For the ratios of MDMA‐to‐MDA, the differences in the means were not statistically significant.

**TABLE 3:** Concentrations of MDA present in blood by case type (mg/L). MDMA‐MDA ratios are given.

Case type	No. of cases	Min.	Max.	Mean^a^	SD	%RSD	Median	Mean ratio
Coronial	33	0.01	0.20	0.06	0.05	97.0	0.04	29.2
Hospitalized drivers	47	0.01	0.21	0.03	0.03	115.6	0.02	14.8
Impaired drivers	31	0.01	0.07	0.03	0.02	58.3	0.02	15.8
Total DUID	78	0.01	0.21	0.03	0.03	92.7	0.02	15.2

^a^ A significantly higher mean concentration of MDA was observed in Coronial cases compared to DUID cases (*p* = 0.0037). The difference between mean MDMA‐MDA ratios was not statistically significant (*p* = 0.9287).

#### 3.3.4 Co‐use of other drugs in DUID cases

In the MDMA‐positive DUID cases, other drugs and/or alcohol were detected in 89% of cases (*n* = 168 of 189). Between 2013 and 2017, MDMA was not quantified in DUID cases, so four (three in 2014 and one in 2015) were excluded from consideration of co‐use. The mean number of co‐used drugs in DUID cases was 1.9 (range: 1–6, median: 2.0). For DUID cases, the most commonly co‐used substance was cannabinoids (*n* = 108), followed by methamphetamine (*n* = 75) and opioids (*n* = 39), see Figure S5 (top). Although alcohol had also been used by some drivers, it is not included among the co‐used substances listed on Figure S5 (top) because only 110 drivers were analyzed for evidence of alcohol use in the lab (as per Police request).

Of the 110 drivers tested for alcohol (58% of the whole cohort), 77 (41% of the whole cohort) tested negative for alcohol (<10 mg/100 mL), and the mean alcohol concentration in the remaining 33 DUID cases was 70 ± 46 mg/100 mL (range: 8–191, median: 66 mg/100 mL). The drink‐drive alcohol limit in blood in NZ is 50 mg/100 mL and 22 drivers (13% of the co‐using cohort, 0.2% of all DUID cases) were over this limit. Of the 33 DUID cases where alcohol and MDMA were detected, only 10 drivers tested negative for other drugs.

### 3.4 Drug sample results

Of the 191 MDMA drug samples obtained from NZ Police or Customs, 138 were found to contain MDMA at a concentration (>LLOQ, 0.12 mg/L) that could be reliably measured (i.e., with more than one measurement) as shown in Table 4. Overall, our results show that capsules had the highest overall purity, and tablets the lowest (see Figure S7). There was a significant difference in percentage purity of MDMA between sample types (see Table 4). The identity and concentration of diluents and adulterants were not included in this study. MDA was not detected in any of the seized drug samples.

**TABLE 4:** Percentage purity of MDMA in the different drug sample types.^a^

Sample type	No. of samples	Min.	Max.	Mean	SD	%RSD	Median
Capsules	22	71	101	87	8.4	9.6	88
Powders/crystals	79	38	99	82	14.2	17.4	86
Tablets	37	17	59	40	10.7	26.6	43
Overall	138	17	101	71	22.8	31.9	82

^a^ Some drugs samples had no detected MDMA (*n* = 8 powders/crystals) or MDMA was present but at <LLOQ (*n* = 8 powders/crystals and *n* = 2 capsules). There was a significant difference in percentage purity between capsules versus tablets and powders/crystals versus tablets (*p* < 0.0001).

## 4 DISCUSSION

This study of MDMA‐positive DUID and Coroner's cases from NZ provides novel data on the concentrations of MDMA and MDA measured in these case types and the demographics associated with them. A comparison with previously published studies can be found in Tables 5, 6, 7.

**TABLE 5:** Summary of the concentrations (mg/L) reported in previous MDMA‐positive fatalities. A “–” indicates the data was not reported.

Location	Dates	MDMA cases^a^	Min.	Max.	Mean	SD	Median	MDA cases^a^	Min.	Max.	Mean	SD	Median	Mean ratio	Ref.
This study	2010–2022	73	0.01	9.30	0.88	1.59	0.20	33	0.01	0.20	0.06	0.05	0.04	29.2	N/A
San Francisco (Armenian and Rodda)	2000–2019	105	0.01	5.60	0.59	0.93	0.29	45	0.01	10.9	0.32	0.36	0.02	–	[26]
Australia (Roxburgh and Lappin)	2000–2018	342	0.01	64.0	–	–	0.45	–	–	–	–	–	–	–	[30]
Taiwan (Lin et al.)	2001–2008	59	0.08	40.4	3.62	6.57	2.02	41	0.05	1.81	0.18	0.28	0.12	26.3	[29]
New York (Gill et al.)	1997–2000	21	<0.10	3.70	0.89	0.88	0.60	18	<0.10	0.80	0.16	0.19	0.10	7.74	[27]
Spain (Lora‐Tamayo et al.)	1993–1995	10	0.03	8.00	1.51	2.57	0.38	8	0.04	1.20	0.32	0.39	0.17	4.14	[31]
The Netherlands (Verschraagen et al.)	1997–2004	51	0.06	84.0	5.50	–	1.60	46	0.01	3.80	0.29	–	0.13	–	[28]

^a^ Number of cases where MDMA or MDA was quantified.

### 4.1 Demographics

Similar ethnicity patterns were observed in other studies of MDMA‐positive deaths, for example, in a study of MDMA‐positive cases from San Francisco, the ethnicities were 40% White, 43% Black, 11% Asian, 6% Hispanic, and 1% Pacific Peoples [26]. For a similar study from New York, the ethnicities were 86% White and 14% Hispanic [27]. Ethnicity was not reported in the other studies mentioned in Table 5.

For the Coronial cases in this study, males were overrepresented at 74% (*n* = 97) and females were underrepresented at 26% (*n* = 34). In the San Francisco study, a similar overrepresentation of males was seen at 87%, and this has been reported elsewhere (Australia 81%; New York 82%; Taiwan 66%; Spain 100%; Sri Lanka 100%; The Netherlands 73%; Portugal 100%) [27, 28, 29, 30, 31, 32, 33]. Both MDMA‐toxicity fatalities in Italy were female [13], and 75% were male in the United Kingdom [34]. Gender was not recorded for the DUID cases in this study.

For the Coronial cases in this study, the age range was 16–70 years (mean: 31 ± 12.9, median: 28). This is a similar range to previous studies such as in San Francisco (range: 16–86, mean: 30 ± 10.7, median: 28) [26]. Other studies have a more narrow age range skewed toward younger groups, such as in Australia (range: 15–58, median: 26, mean not given) [30], New York (range: 17–41, mean: 27, median not given) [27], Taiwan (range: 14–46 years, mean: 25 ± 6.6, median: 23) [29], and Spain (range: 17–39, mean: 26 ± 6.6, median: 27) [31]. These differences may be due to the cases selected for each study. For example, the Australian study looked at deaths where the Coroner considered MDMA to be the underlying cause of death [30] rather than all causes of death. In addition, some previous studies have reviewed cases from the late 1990s and early 2000s, which may not reflect more recent drug use patterns [29, 30].

For the DUID cases in this study, the age range was 15–66 years (mean: 28 ± 9.9, median: 25). Although age is rarely given for MDMA‐positive drivers in the literature, in Norway, the age range was very similar to this study, at 15–65 years (median: 27, mean not given) [35]. In the MDMA‐positive drivers from France, 56% were in the age category 15–24 years [36] compared to 47% in this study. The prevalence of MDMA in younger drivers is likely due to its use being more common in this age group: in New Zealand in 2023/2024, 10.5% of 15–24‐year‐olds reported past‐year use of MDMA, with use declining in those over 35 years [7]. In a recent NZ study of hospitalized drivers, the age range with the highest drug use was 30–39 years at 65%, higher than the 18% (*n* = 34) in this study; however, that study looked at wider drug use and not just MDMA [20].

In this study, the location of the drivers was determined by NZ Police District for 186 DUID cases. The majority of MDMA‐positive DUID cases came from the North Island of NZ (*n* = 121, 64%). This is not consistent with previous wastewater analysis estimating daily MDMA use to be higher in the South Island of NZ [10] or with the highest self‐reported MDMA use per capita being in the Southern region [7]. Although overall MDMA use among drivers in this study was higher in the North Island, the highest percentage of cases by NZ Police District was Canterbury at *n* = 29 (15%), which is located in the South Island.

### 4.2 Prevalence in DUID cases

In this study overall, 193 of 10,840 drivers were positive for MDMA (1.8%). From 2014 to 2022, the prevalence range for MDMA‐positive years was 0.2–3.8%. This is consistent with a recent study, a reanalysis of 530 hospitalized drivers in NZ, where 4% were positive for MDMA [20].

The small number of MDMA‐positive drivers between 2010 and 2016 likely reflects the worldwide shortage of the drug (driven by a shortage of the precursor safrole starting in 2008 [37]), resulting in a scarcity of the drug in NZ. Production moved to starting from the precursor piperonyl methyl ketone (PMK), revitalizing MDMA production in the years following [38]. There was a steep decline in MDMA‐positive DUID cases in 2021 and 2022. Although we do not know the exact cause of the decline, some possible factors include disruption in the imported MDMA supply and lack of events such as music festivals during the COVID‐19 period.

The prevalence of MDMA in drivers in NZ lies within the range reported in previous studies [18, 19, 39, 40, 41]. It is higher than the prevalence in Australia, where less than 1.0% of drivers typically test positive for MDMA, consistent with the lower use of MDMA in Australia compared to NZ [7]. In Europe, the prevalence of MDMA in drivers appears to be higher than in NZ: in a review of 2369 drivers in Hungary, 196 were positive for MDMA (8.3%) [42]; in a French study, 436 of 12,497 drivers (3.5%) were positive for MDMA [36]; in 7593 hospitalized drivers in Italy, 2.2% were positive for amphetamines (MDMA, amphetamine, MDEA, MDA) [43]; in a study of 376 impaired drivers in the United Kingdom, 8 (2.1%) were positive for MDMA [44], and in 127,000 drivers in Norway, 2569 tested positive for MDMA (2.0%) [35]. These differences cannot be explained by MDMA prevalence in different European countries, as past‐year MDMA use among adults (15–64 years) was as follows: Hungary (0.6%), France (1.8%), Italy (0.8%), and Norway (0.9%) [45]. The United Kingdom past‐year MDMA prevalence in 16–59‐year‐olds was 1.2% [46], and in NZ, it was 4.8% [7]. Differences in methodology such as selection of tests [47], availability of testing instrumentation and case selection (hospitalized, impaired, or randomly stopped drivers) may account for some of the differences in prevalence observed.

### 4.3 Concentrations of MDMA in toxicology cases

#### 4.3.1 Coronial cases

A comparison of the drug concentrations obtained for MDMA‐positive deaths in this study with previous studies reported in the literature is given in Table 5. Overall, the concentrations in this study are within the range reported previously, but the maximum concentrations of MDMA and MDA detected in this study (9.3 and 0.2 mg/L, respectively) are lower than observed by Verschraagen et al. [28] (84.0 and 3.8 mg/L), Roxburgh and Lappin [30] (64.0 mg/L), and Lin et al. [29] (40.4 and 1.8 mg/L). In the MDMA‐toxicity deaths (Table 6), the highest MDMA concentration observed in this study was 6.1 mg/L compared to 84.0 mg/L in the other studies (although there were some high concentrations of MDMA in the “Unascertained” cause of death category in our data). However, this still falls within the range associated with MDMA overdose fatalities [48]. There may also be differences in sample types between studies, for example, peripheral versus central postmortem blood. There is significant overlap between MDMA blood concentrations found in this study in Coronial cases and those found in DUID cases (Coronial range: 0.01–9.30 mg/L, DUID range: 0.01–7.30 mg/L). This is likely due to both types of case featuring MDMA as an incidental finding, where blood concentrations are consistent with normal recreational use, that is, less than 0.1 mg/L [48]. For both types of cases, the most common concentration range was 0.01–0.1 mg/L (47% for Coronial cases and 60% for DUID cases, see Figure S9 and Table S2). In drivers, MDMA can cause impairment in the day(s) following use, when blood concentrations have dropped to lower levels [5]. High concentrations among drivers may overlap with those found in fatal MDMA toxicity cases where the user has developed tolerance [49].

**TABLE 6:** Summary of the concentrations (mg/L) reported in previous MDMA‐toxicity fatalities.

Location	Dates	MDMA cases	Min.	Max.	Mean	SD	Median	MDA cases	Min.	Max.	Mean	SD	Median	Mean ratio	Ref.
This study	2010–2022	5	0.02	3.90	2.62	2.46	2.40	4	0.03	0.2	0.12	0.09	0.13	29.2	N/A
Italy (Politi et al.)	2023	2	1.75	3.70	2.73	1.37	2.73	2	0.08	0.10	0.09	0.01	0.09	29.4	[13]
San Francisco (Armenian and Rodda)	2000–2019	5	0.25	5.60	2.83	2.29	1.99	–	–	–	–	–	–	–	[26]
Australia (Roxburgh and Lappin)	2000–2018	14	0.04	64.00	–	–	1.20	–	–	–	–	–	–	–	[30]
Taiwan (Lin et al.)	2001–2008	16	0.48	16.53	3.76	4.41	2.78	12	0.05	0.30	0.16	0.07	0.15	30.8	[29]
New York (Gill et al.)	1997–2000	2	0.60	3.70	2.15	2.19	2.15	2	0.10	0.80	0.45	0.49	0.45	5.31	[27]
United Kingdom (Elliott)	2005	4	1.14	7.25	3.25	2.72	2.31	3	0.02	0.21	0.11	0.10	0.09	38.8	[34]
Portugal (Castro et al.)	2020	1	2.28					1	0.05					46.5	[33]
Sri Lanka (Gunawardane et al.)	2018	4	0.75	3.89	1.67	1.49	1.02	–	–	–	–	–	–	–	[32]
The Netherlands (Verschraagen et al.)	1997–2004	24	1.20	84.00	9.13	17.02	3.65	24	0.04	3.80	0.40	0.76	0.19	27.3	[28]

#### 4.3.2 DUID cases

MDMA has been shown to affect driving ability [16, 50], but there are few papers reporting concentrations of MDMA in drivers in the literature. It can be seen from Table 7 that the range of concentrations of MDMA found in drivers in NZ is higher than those reported in other countries, but the mean MDMA concentration (0.23 mg/L) is similar to France (0.22 mg/L) [36] and the United Kingdom (0.26 mg/L) [44]. Comparisons with other studies are hampered by the lack of reported data. Of note, the Norway study found no correlation between blood concentration of MDMA and impairment as measured by an impairment test [35]. Only one other study of MDMA‐positive drivers reported a series of MDA values (Hungary), and the range was similar to that reported in NZ [42]. There is no per se limit for MDMA or MDA in NZ for drivers; however, the average MDMA concentration reported in this study (0.23 mg/L) is consistent with averages previously reported for DUID cases of 0.26–0.34 mg/L [48, 51].

**TABLE 7:** Summary of the concentrations of MDMA and MDA (mg/L) reported in previous studies of drivers.

Location	Dates	Type of driver	MDMA cases^a^	Min.	Max.	Mean	SD	Median	MDA cases^a^	Min.	Max.	Mean	SD	Median	Ref.
This study	2010–2022	Hospital, impaired	186	0.01	7.30	0.23	0.57	0.08	78	0.01	0.21	0.03	0.03	0.02	N/A
Hungary (Institóris et al.)	2016–2018	Impaired	171	0.05	0.98	–	–	0.10	86	0.05	0.20	–	–	0.22	[42]
Norway (Heide et al.)	2000–2022	Impaired, hospital, random	2569	0.01	3.87	–	–	0.18	–	–	–	–	–	–	[35]
France (Le Daré et al.)	2010–2018	Hospital, checkpoints, random	436	0.01	1.92	0.22	0.25	0.14	–	–	–	–	–	–	[36]
United Kingdom (Burch et al.)	2010–2011	Impaired	8	0.14	0.46	0.26	–	0.23	1	0.05					[44]

^a^ At festivals and events.

### 4.4 Co‐use of other drugs

#### 4.4.1 Coronial cases

In the MDMA‐positive Coronial cases in this study, other drugs and/or alcohol were detected in 86% (*n* = 113) of cases. This is similar to previous studies of fatalities (range: 73–100%). For example, in a study of MDMA‐positive cases from San Francisco, other drugs and/or alcohol were present in 91% of cases. The top three co‐ingested drugs were other amphetamines (57%), cocaine (48%), and ethanol (40%) [26]. In an Australian study, it was 85% of cases, and the most commonly co‐used drugs were methamphetamine (44%), alcohol (43%), and opioids (30%) [30]. For a series of cases from Taiwan, 73% were positive for other drugs and/or alcohol, with the most commonly co‐used drugs being ketamine (42%), sedatives (17%), and ethanol (17%) [29]. In a study from New York, the figure was 86%, and the most commonly co‐used substances were ethanol, opiates, and cocaine (all 32%) [27]. In a Spanish study, all of the MDMA‐positive cases reported contained other drugs and/or alcohol with the most common being ethanol (70%), followed by amphetamine and 3,4‐methylenedioxy‐*N*‐ethylamphetamine (MDEA) (50% each) [31]. In a series of cases from The Netherlands, no overall value for co‐use was given, but the most common three drugs were alcohol (40%), cocaine (39%), and benzodiazepines (19%) [28]. New Zealand is similar to New York, Spain, and The Netherlands in having alcohol as the most commonly co‐used drug in Coronial cases.

#### 4.4.2 DUID cases

Figure S5 shows the three most commonly co‐used substances in this study. Cannabis is often found in the top three co‐used drugs in MDMA‐positive DUID cases. For example, in The Netherlands, the most common three drugs used by drivers alongside MDMA were cannabis (35%), alcohol (32%), and cocaine (28%) [28], and in Norway, drivers had used amphetamine (63%), cannabis (48%), and clonazepam (33%) alongside MDMA [35]. The high position of cannabis in these studies likely reflects its worldwide prevalence [52].

In 20% (*n* = 33) of the DUID cases in this study that were positive for alcohol and/or other drugs, alcohol was detected. However, this is not a true reflection of the prevalence of co‐use of MDMA and alcohol in NZ drivers because not all DUID cases are tested for alcohol in the laboratory. As mentioned earlier, this is likely because the driver already passed a roadside breathalyzer test. In the case of some drivers, alcohol may have been below the legal limit (250 μg/L in breath) but not a 0 result. In a study from Victoria, Australia, 0.3% of drivers were positive for MDMA and also over the proscribed alcohol limit, compared to 0.2% in this study [41].

### 4.5 Drug sample results

In this study, the median percentage purity was 43% for MDMA tablets. This is higher than reported for other countries including the United Kingdom (median: 25% and 30% for two separate studies) and Belgium (median: 39%) (see Table 8) but the range of purities for tablets in this study is more narrow than others (17–58% compared to 1–73% in the literature) [4, 15, 53] (see Figure S8). However, the number of tablet samples tested in this study (*n* = 37) was small compared to others [15, 53]. There are no published studies reporting MDMA purities outside of NZ [3] for drug samples other than tablets.

**TABLE 8:** Percentage purity of MDMA in tablet samples reported previously in the literature. A box plot of the datasets is shown in Figure S8.

Location	Dates	No.	Percentage purity				Ref.
			Min.	Max.	Mean	Median	
This study	2018–2019	37	17	59	40	43	N/A
United Kingdom (Couchman et al.)	2001–2008	412	1	72	–	25	[15]
Belgium (Deconinck et al.)	2016–2017	199	18	73	39	39	[53]
United Kingdom (Naqi et al.)	–	11	14	42	28	30	[4]

Information on the higher purity of MDMA capsules found in NZ compared to other sample types could be disseminated through harm‐reduction initiatives [8]. However, there is not a straightforward link between purity and harm, as low‐purity MDMA samples may contain harmful adulterants [54].

### 4.6 Limitations

There are a number of limitations to this study. For the Coronial data, we may not have captured all deaths where MDMA was present during the study period, as not all deaths are referred to a Coroner. Of those referred to a Coroner, not all have toxicology testing undertaken on them. Although some deaths were reported as being due to MDMA toxicity, there were some cases where the cause of death was unascertained in this study, which may have been due to MDMA overdose. We also cannot determine the exact role MDMA may have played in some of the other causes of death [28]. We have reported concentrations of MDMA and MDA in Tables 2 and 3, but these drugs may exhibit postmortem redistribution [48], which should be considered when interpreting the results. We have assumed that MDA was present as a metabolite of MDMA, but it is also a drug in its own right that may be found alongside MDMA [49]. We did not distinguish between the enantiomers of MDMA in this work [55]. For the drug sample data, we did not measure a dose of MDMA per tablet or capsule, which limited the comparisons we could make with previous literature. We also did not examine adulterants or diluents in the seized drug samples [54]. For DUID cases that tested negative for alcohol, we cannot exclude the possibility that alcohol was present at the time of the crash, but a delay before blood sampling resulted in metabolism of the alcohol.

## 5 CONCLUSION

In summary, this paper provides toxicological and demographic data for MDMA‐positive fatalities and MDMA‐positive drivers in NZ between 2010 and 2022. Alongside, we have provided purity data for a range of MDMA‐containing drug samples (powders/crystals, capsules, and tablets). The demographics of MDMA users were comparable to those reported in previous studies. Co‐use of alcohol and/or other drugs was a common finding. The prevalence of MDMA in drivers is similar to that reported in previous studies. There is no obvious overall trend in MDMA use in NZ apparent from the data, with MDMA use increasing in drivers followed by a decrease over the study period, but increasing in Coronial cases. Our data also show that capsules have the highest purity of MDMA compared to pills or powders/crystals. We envisage the data presented in this paper being of use for interpretation by forensic toxicologists, and decision‐making by law enforcement and drug policymakers.

## CONFLICT OF INTEREST STATEMENT

The authors have no conflicts of interest to declare.

## Supporting information


Figure S1.

Figure S2.

Figure S3.

Figure S4.

Figure S5.

Figure S6.

Figure S7.

Figure S8.

Figure S9.

Table S1.

Table S2.

## Data Availability

The data that support the findings of this study are available on request from the corresponding author. The data are not publicly available due to privacy or ethical restrictions.
