# Supplementary figures and images for: Trends in toxicological findings and drug seizures of MDMA in New Zealand from 2010 to 2022
Source: J Forensic Sci. 2026 Feb 12;71(3):1338–50. doi: 10.1111/1556-4029.70284 (PMC13139824; doi:10.1111/1556-4029.70284)

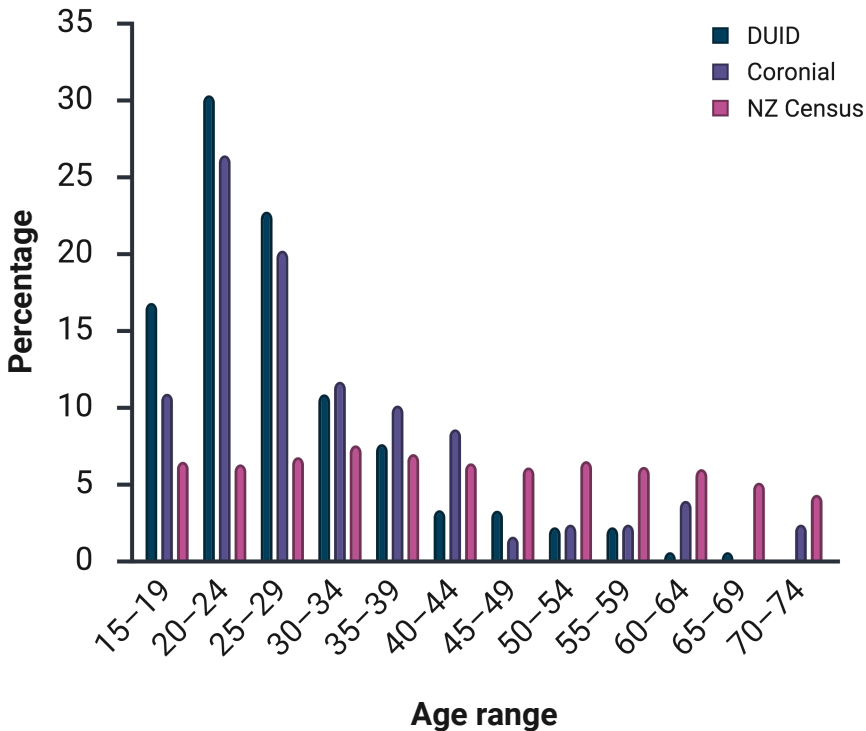

Supplement: Supplementary file 1 — Figure S1. [file JFO-71-1338-s003.pdf]

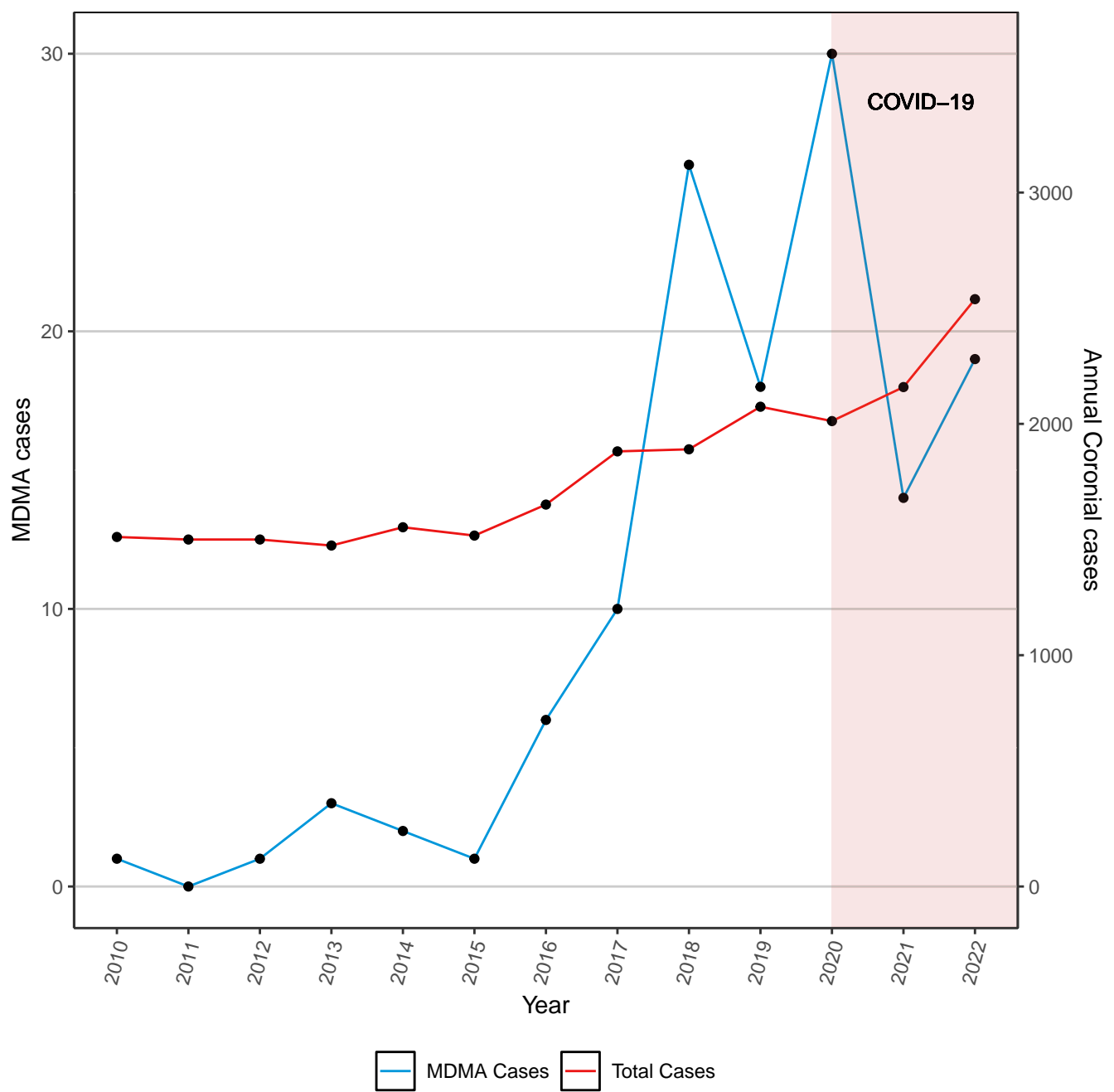

Supplement: Supplementary file 2 — Figure S2. [file JFO-71-1338-s004.pdf]

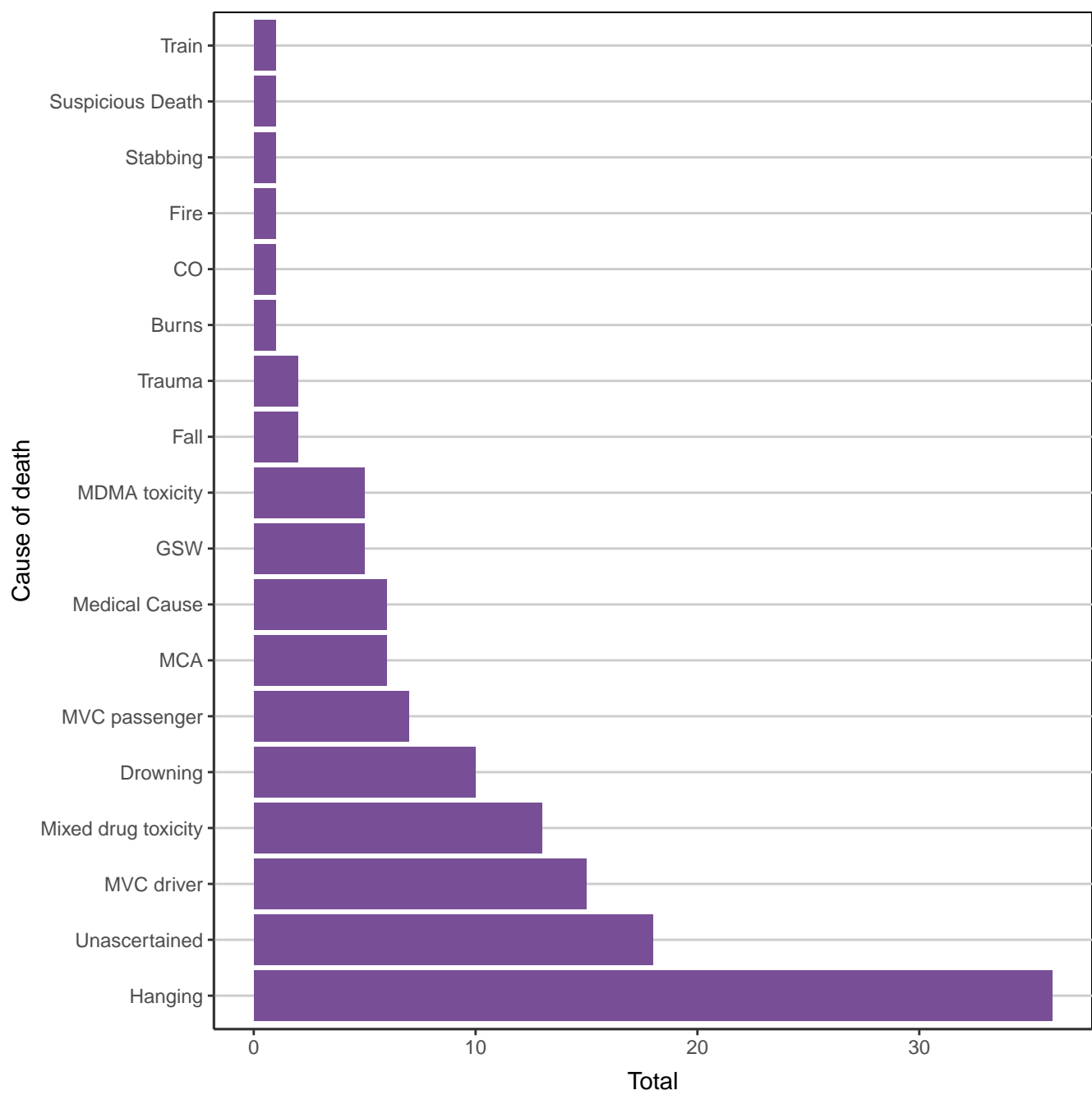

Supplement: Supplementary file 3 — Figure S3. [file JFO-71-1338-s007.pdf]

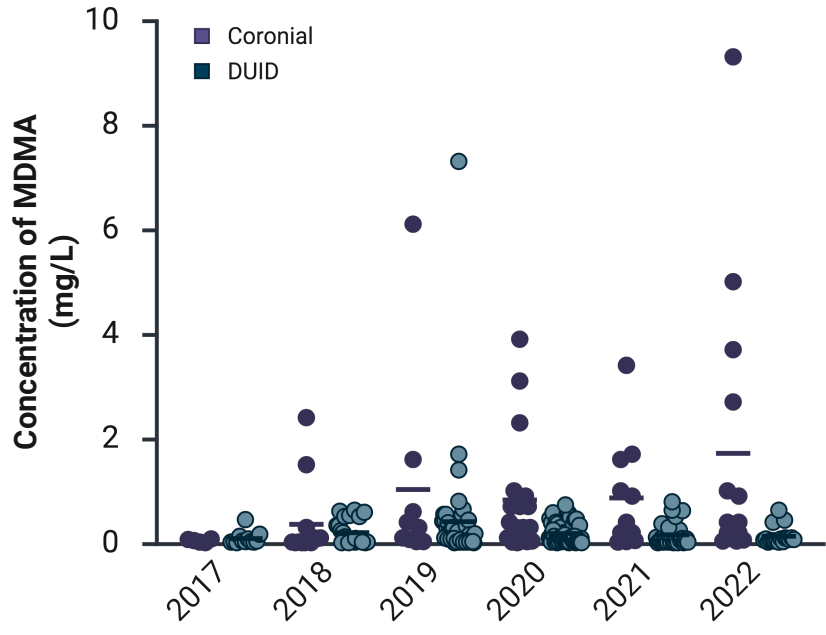

Supplement: Supplementary file 4 — Figure S4. [file JFO-71-1338-s005.pdf]

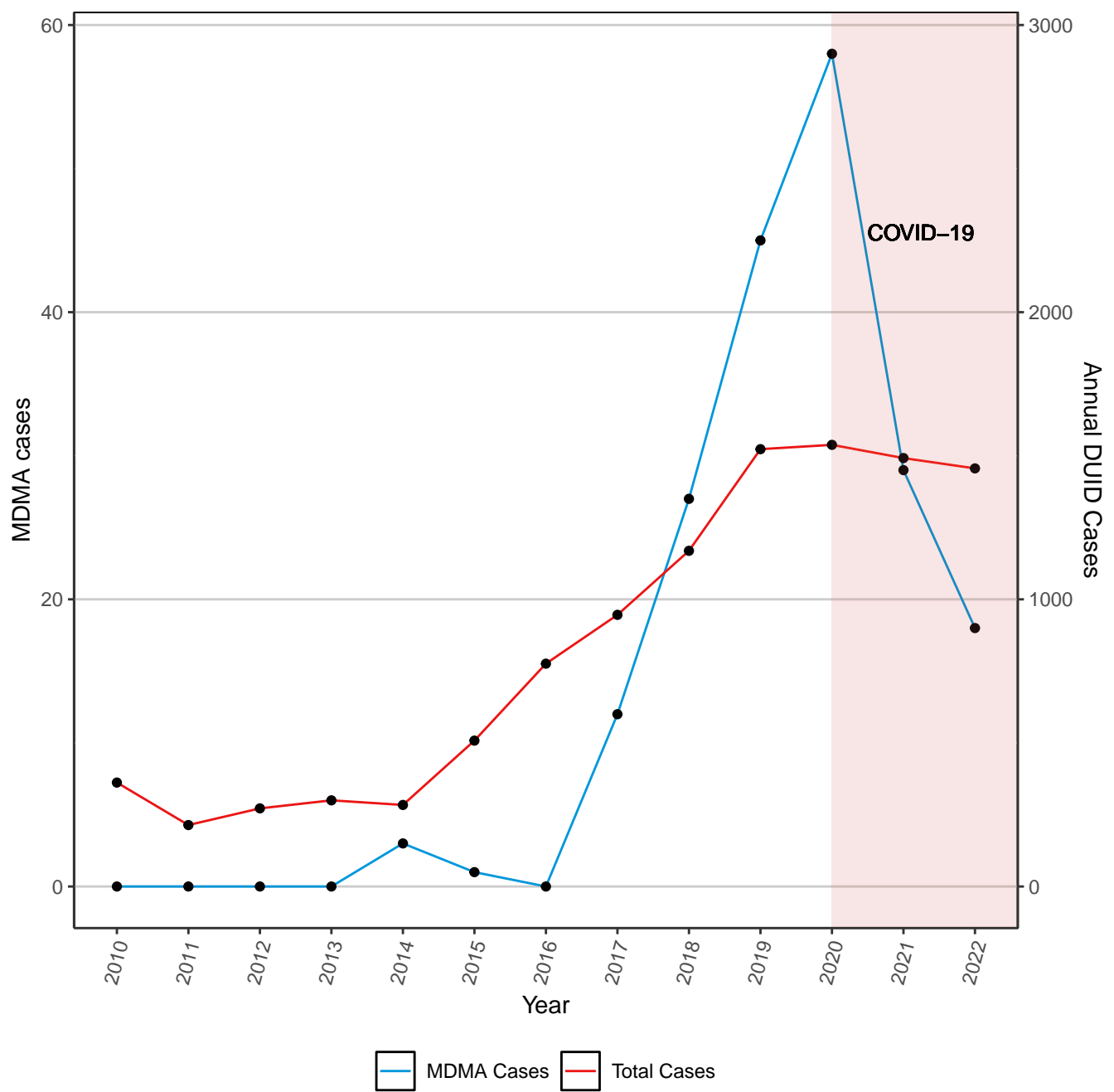

Supplement: Supplementary file 6 — Figure S6. [file JFO-71-1338-s010.pdf]

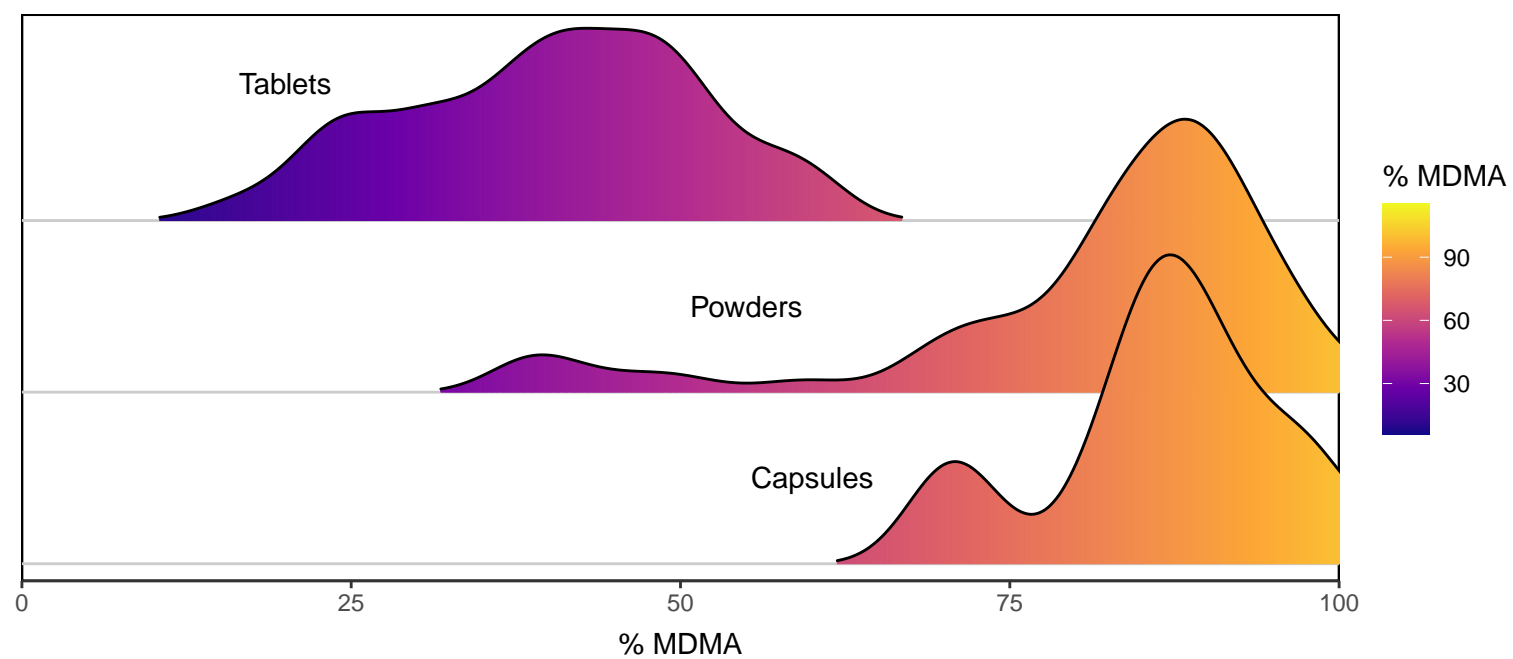

Supplement: Supplementary file 7 — Figure S7. [file JFO-71-1338-s001.pdf]

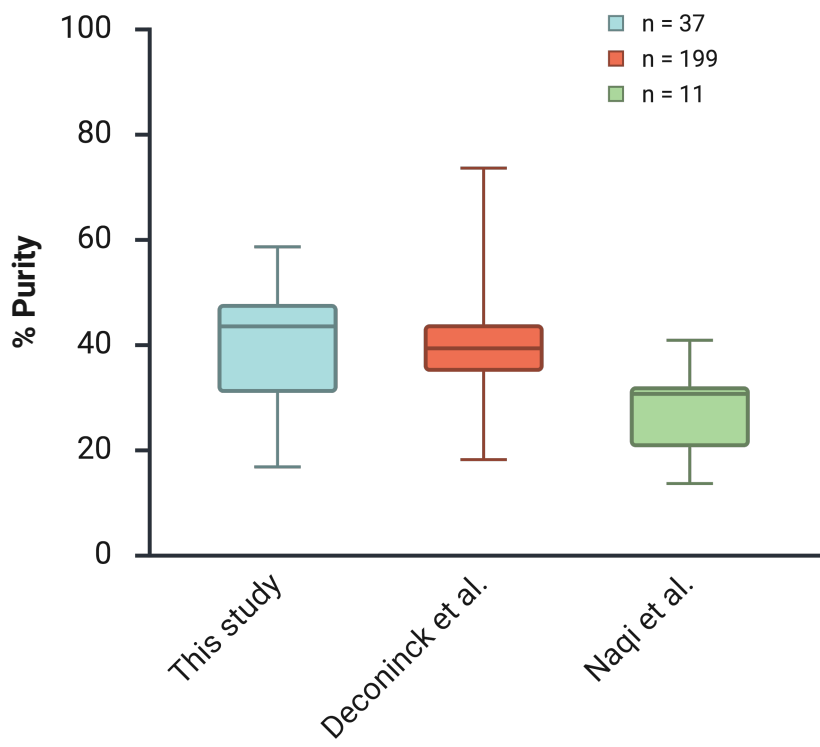

Supplement: Supplementary file 8 — Figure S8. [file JFO-71-1338-s006.pdf]

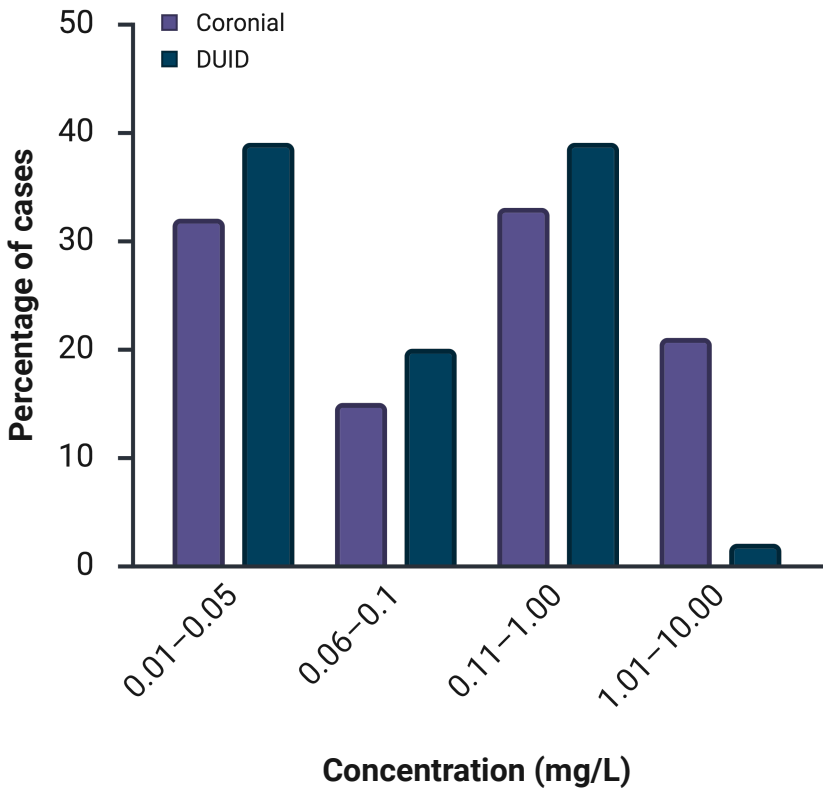

Supplement: Supplementary file 9 — Figure S9. [file JFO-71-1338-s002.pdf]
