# Supplementary material for: Trends in toxicological findings and drug seizures of MDMA in New Zealand from 2010 to 2022
Source: J Forensic Sci. 2026 Feb 12;71(3):1338–50. doi: 10.1111/1556-4029.70284 (PMC13139824; doi:10.1111/1556-4029.70284)
Supplement: Supplementary file 5 — Figure S5. [file JFO-71-1338-s008.pdf]

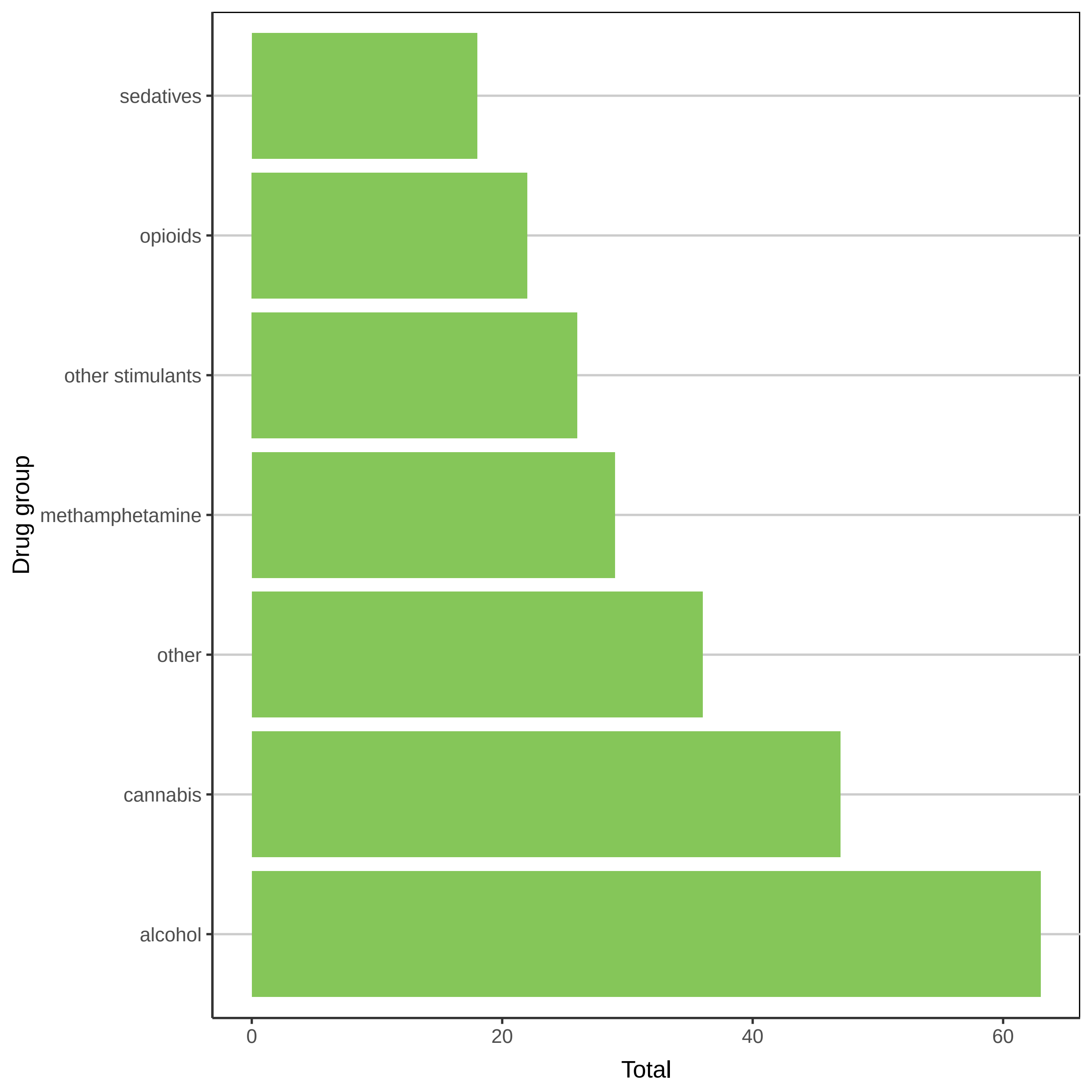

sedatives
opioids
other stimulants
methamphetamine
other
cannabis
alcohol
Drug group
0
20
40
60
Total

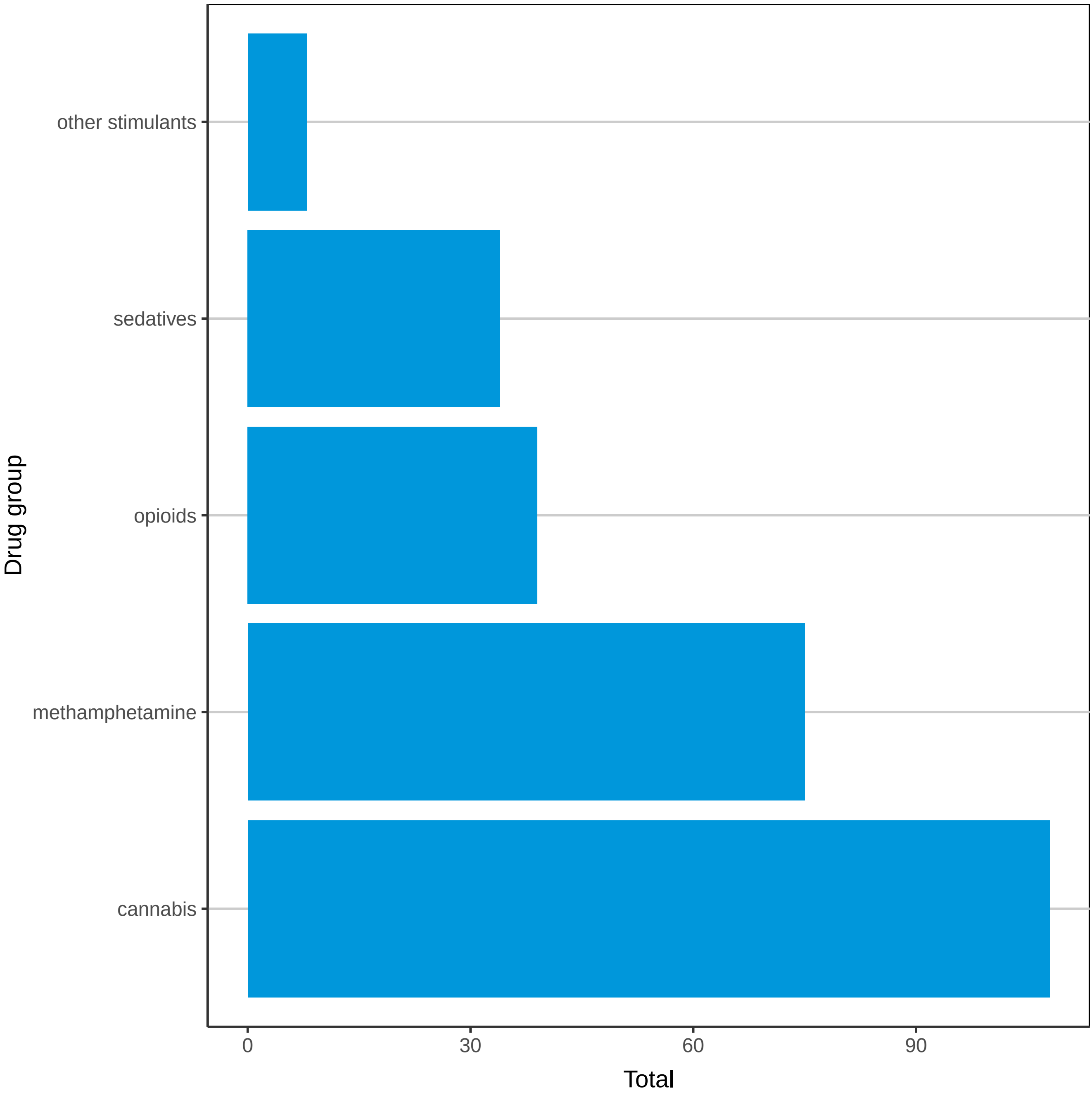
other stimulants
sedatives
opioids
methamphetamine
cannabis
Drug group
0
30
60
90
Total
