# Supplementary material for: Trends in toxicological findings and drug seizures of MDMA in New Zealand from 2010 to 2022
Source: J Forensic Sci. 2026 Feb 12;71(3):1338–50. doi: 10.1111/1556-4029.70284 (PMC13139824; doi:10.1111/1556-4029.70284)
Supplement: Supplementary file 10 — Table S1. [file JFO-71-1338-s011.docx]

## TABLE S1 Average concentration of MDMA by case type and year.

| Year | MDMA-positive Coronial cases | Average MDMA concentration (mg/L) | MDMA-positive DUID cases | Average MDMA concentration (mg/L) |
| --- | --- | --- | --- | --- |
| 2017 | 5 | 0.05 | 12 | 0.09 |
| 2018 | 13 | 0.36 | 26 | 0.20 |
| 2019 | 9 | 1.03 | 45 | 0.41 |
| 2020 | 18 | 0.82 | 56 | 0.18 |
| 2021 | 11 | 0.86 | 29 | 0.16 |
| 2022 | 14 | 1.72 | 18 | 0.13 |
| Total | 73 |  | 186 |  |
