# Supplementary material for: Trends in toxicological findings and drug seizures of MDMA in New Zealand from 2010 to 2022
Source: J Forensic Sci. 2026 Feb 12;71(3):1338–50. doi: 10.1111/1556-4029.70284 (PMC13139824; doi:10.1111/1556-4029.70284)
Supplement: Supplementary file 11 — Table S2. [file JFO-71-1338-s009.docx]

## TABLE S2 Distribution of concentrations of MDMA by case type.

| Range (mg/L) | Coronial (*n*) | Coronial (%) | DUID (*n*) | DUID (%) |
| --- | --- | --- | --- | --- |
| 0.01–0.05 | 23 | 32 | 73 | 39 |
| 0.06–0.10 | 11 | 15 | 38 | 20 |
| 0.11–1.00 | 24 | 33 | 72 | 39 |
| 1.00–10.0 | 15 | 21 | 3 | 2 |
| Total | 73 |  | 186 |  |
